# Model-informed dose optimization of mycophenolic acid in pediatric kidney transplant patients

**DOI:** 10.1007/s00228-024-03743-0

**Published:** 2024-08-17

**Authors:** Astrid Heida, Nynke G. L. Jager, Rob E. Aarnoutse, Brenda C. M. de Winter, Huib de Jong, Ron J. Keizer, Elisabeth A. M. Cornelissen, Rob ter Heine

**Affiliations:** 1 Department of Pharmacy, Radboud Institute for Medical Innovation, Radboud University Medical Center, Nijmegen, The Netherlands; 2 Department of Hospital Pharmacy, Erasmus University Medical Center, Rotterdam, The Netherlands; 3 The Erasmus MC Transplant Institute, Erasmus University Medical Center, Rotterdam, The Netherlands; 4 Insight Rx, San Francisco, CA USA; 5 Department of Pediatric Nephrology, Radboud University Medical Center, Amalia Children’s Hospital, Nijmegen, The Netherlands

**Keywords:** Mycophenolic acid, Pediatrics, Population PK, NONMEM, Kidney, Transplantation

## Abstract

**Purpose:**

We aimed to develop and evaluate a population PK model of mycophenolic acid (MPA) in pediatric kidney transplant patients to aid MPA dose optimization.

**Methods:**

Data were collected from pediatric kidney transplant recipients from a Dutch academic hospital (Radboudumc, the Netherlands). Pharmacokinetic model-building and model-validation analyses were performed using NONMEM. Subsequently, we externally evaluated the final model using data from another academic hospital. The final model was used to develop an optimized dosing regimen.

**Results:**

Thirty pediatric patients were included of whom 266 measured MPA plasma concentrations, including 20 full pharmacokinetic (PK) curves and 24 limited sampling curves, were available. A two-compartment model with a transition compartment for Erlang-type absorption best described the data. The final population PK parameter estimates were K_tr_ (1.48 h^−1^; 95% CI, 1.15–1.84), CL/F (16.0 L h^−1^; 95% CI, 10.3–20.4), V_c_/F (24.9 L; 95% CI, 93.0–6.71E25), V_p_/F (1590 L; 95% CI, 651–2994), and Q/F (36.2 L h^−1^; 95% CI, 9.63–74.7). The performance of the PK model in the external population was adequate. An optimized initial dose scheme based on bodyweight was developed. With the licensed initial dose, 35% of patients were predicted to achieve the target AUC, compared to 42% using the optimized scheme.

**Conclusion:**

We have successfully developed a pharmacokinetic model for MPA in pediatric renal transplant patients. The optimized dosing regimen is expected to result in better target attainment early in treatment. It can be used in combination with model-informed follow-up dosing to further individualize the dose when PK samples become available.

**Supplementary Information:**

The online version contains supplementary material available at 10.1007/s00228-024-03743-0.

## Introduction

Mycophenolic acid (MPA), the active compound of the prodrug mycophenolate mofetil (MMF), is prescribed to prevent allograft rejection in pediatric kidney transplant patients. Its pharmacokinetics (PK) are characterized by a large inter- and intra-individual variability, particularly in the early post-transplant period [1–3]. The total MPA plasma exposure, i.e., the area under the concentration versus time curve (AUC_0–12 h_) is correlated with efficacy and toxicity. When plasma exposure falls outside the therapeutic window of AUC_0–12 h_ 30–60 mg h L^−1^, the risks of graft rejection or the risk of toxicity increase significantly [4–6]. Therefore, therapeutic drug monitoring (TDM) is recommended to ensure exposure remains within the defined therapeutic window [4, 7]. TDM of MPA is complicated by the fact that the trough concentration of MPA is poorly correlated with the AUC [7]. Therefore, multiple samples within one dosing interval need to be obtained to reliably estimate the AUC [4, 7].

The licensed dose of MMF for pediatric patients is 1200 mg m^−2^ day^−1^ in two divided doses, based on studies in pediatric patients co-treated with cyclosporine and steroids [8–10]. It is known that cyclosporine decreases MPA exposure by inhibiting the enterohepatic recirculation of MPA [4]. Hence, a starting dose of 1200 mg m^−2^ a day might be too high for patients receiving a combination of MPA and tacrolimus without cyclosporine. In addition, it is known that body surface area is not an ideal predictor for pharmacokinetics in children as it may deviate from the true relationship between body size and pharmacokinetics [11, 12].

A more personalized approach for the starting dose of MPA after kidney transplantation may result in better pharmacokinetic target attainment early in treatment. Integrating factors that influence MPA exposure may help to predict the optimal MPA starting dose for the individual patient. Such an optimized starting dosing regimen can be developed using a population PK model. After administration of the first doses of MPA, further dose individualization can be guided by measured drug concentrations, i.e., by TDM, or model-informed precision dosing (MIPD). To enable MIPD in clinical practice, the availability of an appropriate PK model is pivotal [13, 14]. Therefore, our aim was to develop and validate a population PK model of MPA in pediatric kidney transplant patients to serve MPA dose optimization, both to develop an optimized starting dose for MPA as well as for TDM-based MIPD thereafter.

### Methods

#### Study population and clinical data collection

Patient and treatment data from pediatric kidney transplant recipients treated with MMF including routine TDM, between June 2016 and April 2023 at the Amalia Children’s Hospital (Radboudumc, Nijmegen, The Netherlands) were used. The Ethical Review Board Oost-Nederland waived necessity for approval as TDM is considered routine clinical care. Data of patients that did not provide consent for the use of their data were not collected. Pediatric kidney transplant patients receiving oral MMF (Cellcept®) in combination with tacrolimus or everolimus were included if at least one full PK curve (approximately 8 samples at *t* = 0, *t* = 1, *t* = 2, *t* = 3, *t* = 4, *t* = 6, *t* = 8, and *t* = 12 h), or limited PK curve (3 samples at *t* = 0, *t* = 0.5, and *t* = 2 h) was available. Routine clinical practice consists of patients starting on 1200 mg m^−2^ of MMF (CellCept®) a day, and after 2 weeks the dose was reduced to 600 mg/m^2^ a day. Subsequent dose adjustments were based on TDM at the discretion of the treating physician. For the model development dataset, the following data were collected: timing of dosing and sample collection, age, gender, weight, height, albumin, creatinine, immunosuppressive comedication, and time after transplantation. Body surface area was calculated with the Dubois formula using patients’ weights and heights [15]. All MPA plasma concentrations were quantified using a validated Enzyme Multiplied Immunoassay Technique (EMIT) assay (Mycophenolic Acid Assay, Roche, Basel, Switzerland) operated on a Cobas type c502 System according to the manufacturer’s guidelines.

#### Model development

PK model-building and model-evaluation analyses were performed with the software package NONMEM version 7.4.1 (ICON plc, Dublin, Ireland) using the model development dataset. All population PK analyses were performed using the first-order conditional estimation method with interaction (FOCE + I). In the dataset, the dose was entered in MMF equivalents (milligrams of MMF) and the dependent variable (concentration units) was entered as MPA equivalents (milligrams of MPA).

To describe the absorption process, Erlang-type absorption was used [16]. The Erlang distribution serves as the analytical solution for a series of compartments located between the dosing and the central compartment [17]. The optimal number of sequential compartments was determined by gradually adding compartments until no further improvement was observed.

During model building, linear pharmacokinetics were assumed. Because no data on absolute bioavailability (F) was available, the model was parameterized as apparent clearance (CL/F) and apparent volumes of distribution (CL/F). To account for changes in PK as a result of body size, all volume and flow parameters were allometrically scaled to a total body weight of 70 kg [18]. The allometric coefficients were fixed at 0.75 for flow parameters and 1 for volume parameters and, consequently, at − 0.25 for rate constants.

Inter-individual variability and inter-occasion variability were explored on all structural model parameters and estimated using an exponential model which assumed a log-normal distribution [19]. Residual variability was evaluated using proportional, additive, and combined residual error models. Ninety-five percent confidence intervals were calculated using sampling importance resampling (SIR) [20]. The coefficient of variation (CV) was calculated using the following formula:. $\mathrm{CV}(\%)=\sqrt{\exp(\mathrm\omega^2)-1}\times100\%$ Furthermore, the extent of shrinkage for inter-individual and inter-occasion variability and residual errors was computed [21, 22].

Decreased plasma albumin concentrations are known to be associated with the PK of MPA, as a result of protein binding [23]. Therefore, the association between the individual PK parameter estimates and albumin was screened by evaluation of albumin level plotted against the individual PK parameters. If a relationship was detected between albumin levels and the individual PK parameters, the covariate was normalized by dividing the value by 35 g/L, and the effect of the covariate was explored using linear, power, and exponential functions. A change in objective function value (OFV) of > 3.84 (*p* < 0.05) was necessary to introduce the covariate in the model.

The final model was selected based on physiological plausibility, objective function value, goodness-of-fit plots, and prediction-corrected visual predictive checks.

### External evaluation of the developed model

To evaluate whether our model might be suitable to use for MIPD, we used an external dataset containing patient- and treatment data from pediatric kidney transplant recipients from the Sophia Children’s Hospital (Erasmus MC, Rotterdam, The Netherlands). The ethical review committee of Erasmus MC waived the necessity of approval as therapeutic drug monitoring (TDM) is considered routine clinical care. MPA concentrations were measured using a validated Liquid chromatography-mass—Mass spectrometry (LC–MS) or EMIT assay (EMIT MPA Assay System; Dade Behring, San Jose, CA, USA).

The external dataset contained PK data of 29 children using MMF (CellCept®). Therapy was initiated at a dosage of 1200 mg m^−2^ a day, and after 2 weeks the dose was reduced to 600 mg m^−2^ a day. Subsequent dose adjustments were based on TDM. The data of 11 patients were excluded due to concomitant use of cyclosporin, which is known to affect MPA PK [4]. Characteristics of the included patients are provided in Table 1. The median age of this patient was 15 years (range, 6.4–18), the median weight was 51.1 kg (range, 17.1–86). The time after transplantation was longer for patients in the external dataset (median of 721 days) compared with the patients in the model development dataset. In addition, the patients in the external data set had a higher albumin level (median, 42 g/l). Comedication data were not available, except for information on whether patients were using cyclosporin. The PK data consisted of 81 PK measurements, mainly shortened PK curves of 6 samples obtained before and 0.167, 0.5, 1.5, 2, and 4 h after administration of MMF.

**Table 1:** Study population characteristics and treatment data at the time of the first sample collection. Results are presented as median and range (minimum–maximum). *NA* not available

	Model development dataset	External dataset
Age (years)	13 (4–18)	15 (6.4–18)
Male/female	18/12	6/12
Weight (kg)	38.5 (12.9–79.9)	51.1 (17.1–86)
Height (cm)	149 (95–193)	154 (111–175.4)
Body surface area (m^2^)	1.3 (0.58–2.1)	1.5 (0.72–2.0)
Albumin (g L^−1^)	34 (24–42)	42 (29–49)
Number of observations per patient	9.5 (3–18)	12 (5–12)
Number of occasions per patient	2 (1–6)	2(1–2)
Mycophenolate mofetil daily dose (mg day^−1^)	1000 (500–2000)	500 (250–1000)
Posttransplant time (days)	9.5 (2–3058)	721 (357–2548)
Immunosuppressive comedication		
Tacrolimus	83.3%	NA
Everolimus	16.7%	NA
Prednisone	13.3%	NA

We evaluated the final population PK model by goodness-of-fit plots and a prediction-corrected visual check. For the prediction-corrected visual predictive check, a total of 1000 simulations were performed using the external dataset. Plots of the median, 5th and 95th percentiles and 95% confidence intervals of the simulated concentration–time profiles were generated to visually verify that the percentiles of the observed concentration–time data were contained within the respective confidence intervals. The main focus of our external evaluation was the correspondence of the individually predicted concentrations versus observed concentrations, as these reflect clinical practice where individual AUCs are assessed and dose adjustments are performed based on these AUCs.

### Development of an improved starting dose

Using the developed PK model, we explored improved starting dose regimens. Based on the relationship between dose, clearance, and steady-state AUC (dose = clearance × AUC), we developed a weight-based dosing algorithm to reach the desired target AUC_0−12 h_ (30–60 mg h L^−1^) [24]. We assumed that whole tablets were used, so the required dose for a twice-daily regimen was rounded to 250 mg, as this is the smallest available tablet size. The performance of this dosing regimen to reach therapeutic exposure was then compared with the licensed dosing regimen of 1200 mg m^−2^ divided over 2 daily doses, also rounded to 250 mg. For this purpose, a virtual representative pediatric population of 1000 patients with age of 3 to 18 years from a representative European pediatric population was generated using PopGen (ICRP database), a population simulation tool [25, 26], with a mean albumin value of 33 g L^−1^ (CV, 21%), based on the observed serum albumin concentrations in the model development dataset. Subsequently, we simulated the AUC_0−12 h_ of patients on the licensed dose and on the improved starting dose to compare target attainment.

## Results

### Study population of the model development dataset

**Fig. 1:**
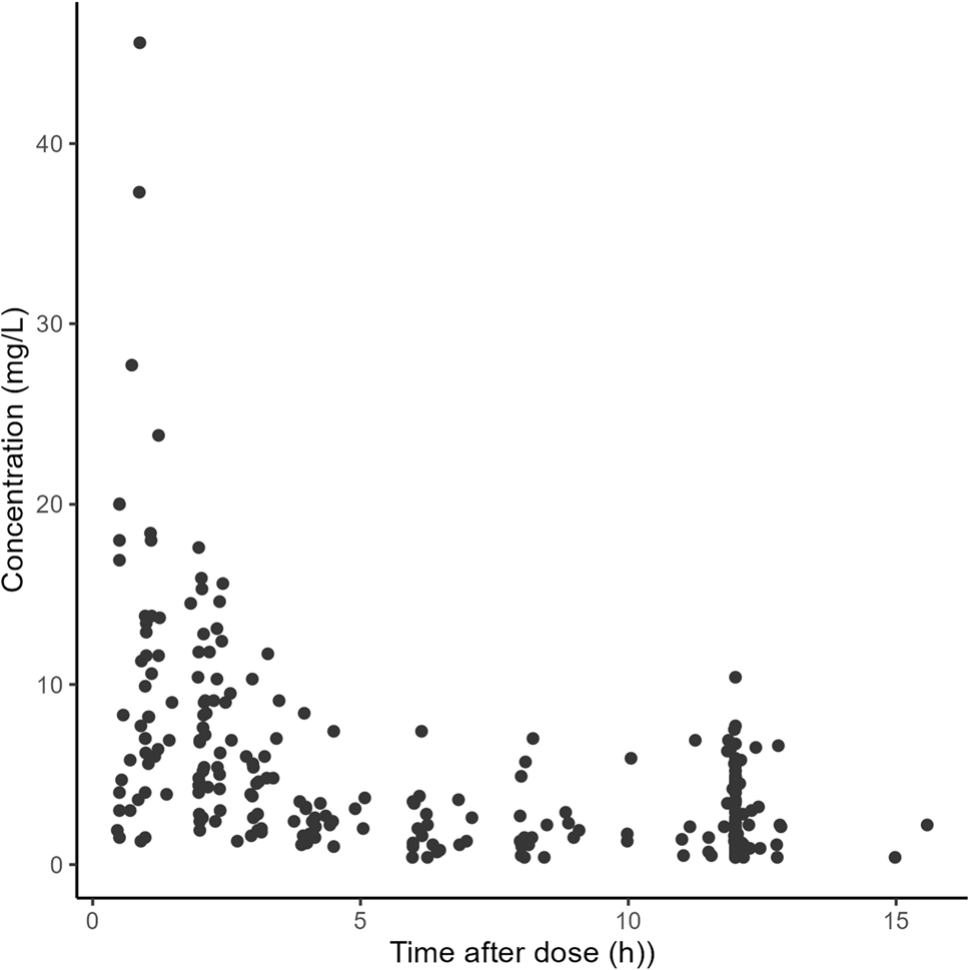
Concentration–time profiles of MPA of the model development dataset. Each dot represents a single measured drug sample

Data from 30 pediatric kidney transplant patients using MMF were used for model development. Patient characteristics are provided in Table 1. The median age was 13 years (range 4 to 18), median weight was 38.5 kg (range 12.9 to 79.9), and median albumin level was 34 g L^−1^ (range 24 to 42). Immunosuppressive comedication included tacrolimus (25 patients) or everolimus (5 patients). These drugs have no known influence on the PK of MMF [27]. PK data consisted of 266 plasma MPA plasma concentrations measured as part of routine clinical therapeutic drug monitoring mostly measured early after transplantation, including 20 full PK curves, 24 limited sampling curves, and 25 trough level measurements (Fig. 1).

### Model development

A two-compartment model with the addition of a single transition compartment for absorption (Erlang-type absorption) best described the data. The addition of more transition compartments did not lead to further improvement of the model. Inter-individual variability could be estimated for clearance, intercompartmental clearance, and central volume of distribution, while inter-occasion variability could be identified for the relative bioavailability (F) [19]. Residual error was best modelled by a proportional error. When plotting the individual estimates for clearance against serum albumin, a clear linear negative relationship was observed, therefore albumin was included as a covariate. The introduction of albumin as a covariate of clearance significantly improved the model (ΔOFV =  − 15.29, *p* < 0.05). The final model equations for the model parameters are displayed in Eqs. 1–5.

**Fig. 2:**
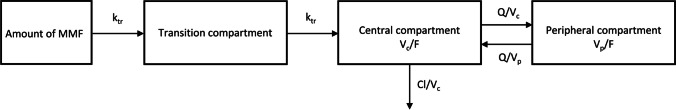
Schematic representation of the final PK model of MPA in pediatric kidney transplant recipients. ktr, transfer rate constant between two absorption compartments; Cl/F, apparent oral clearance; Vc/F, apparent volume of the central compartment; Vp/F, apparent volume of the peripheral compartment; Q, intercompartment clearance

The structural model is schematically depicted in Fig. 2. The final estimates are described in Table 2. The NONMEM control stream of the final model, including the final parameter estimates is provided in the supplementary material.

**Table 2:** Population pharmacokinetic parameters of MPA. Cl/F, apparent oral clearance; V_c_/F, apparent volume of the central compartment; V_p_/F, apparent volume of the peripheral compartment; Q/F, apparent intercompartment clearance; k_tr_, transfer rate constant between two absorption compartments; *F* relative bioavailability

Parameters	Final estimate	95% confidence interval	Shrinkage
Cl/F = θ1 * (body weight/70)^0.75^ *(Albumin/34) ^θ2^			
θ1	16.0 L h^−1^	10.3–20.4	
θ2	− 2.49	− 3.82 to − 1.41	
V_c_/F	24.9 L	6.53–45.8	
V_p_/F	1590 L	651–2994	
Q/F	36.2 L h^−1^	25.8–49.6	
k_tr_	1.48 h^−1^	1.15–1.84	
Inter-individual variability			
Cl/F	38.6% CV	15.38–73.95%	34.6%
V_c_/F	320% CV	109.65–18,819.64%	31.6%
Q/F	63.6% CV	31.75–97.25%	28.0%
Inter-occasion variability			
F	46.1% CV	33.79–60.86%	24.5%
Residual error	47.3% CV	42.47–52.68%	11.7%

1 $$\text{Cl}/\text{F }(\text{L }{\text{h}}^{-1}) = 16.0 \times (\text{weight}/7{0)}^{0.75} \times (\text{albumin}/35{)}^{-2.5}$$

2 $${\text{V}}_{\text{c}}/\text{F }(\text{L})= 24.9 \times (\text{weight}/70)$$

3 $${\text{V}}_{\text{p}}/\text{F }(\text{L})= 1590 \times (\text{weight}/70)$$

4 $$\text{Q}/\text{F }(\text{L }{\text{h}}^{-1}) = 36.2 \times (\text{weight}/70{)}^{0.75}$$

5 $${\text{K}}_{\text{tr}} ({\text{h}}^{-1}) = 1.48 \times (\text{weight}/7{0)}^{-0.25}$$

**Fig. 3:**
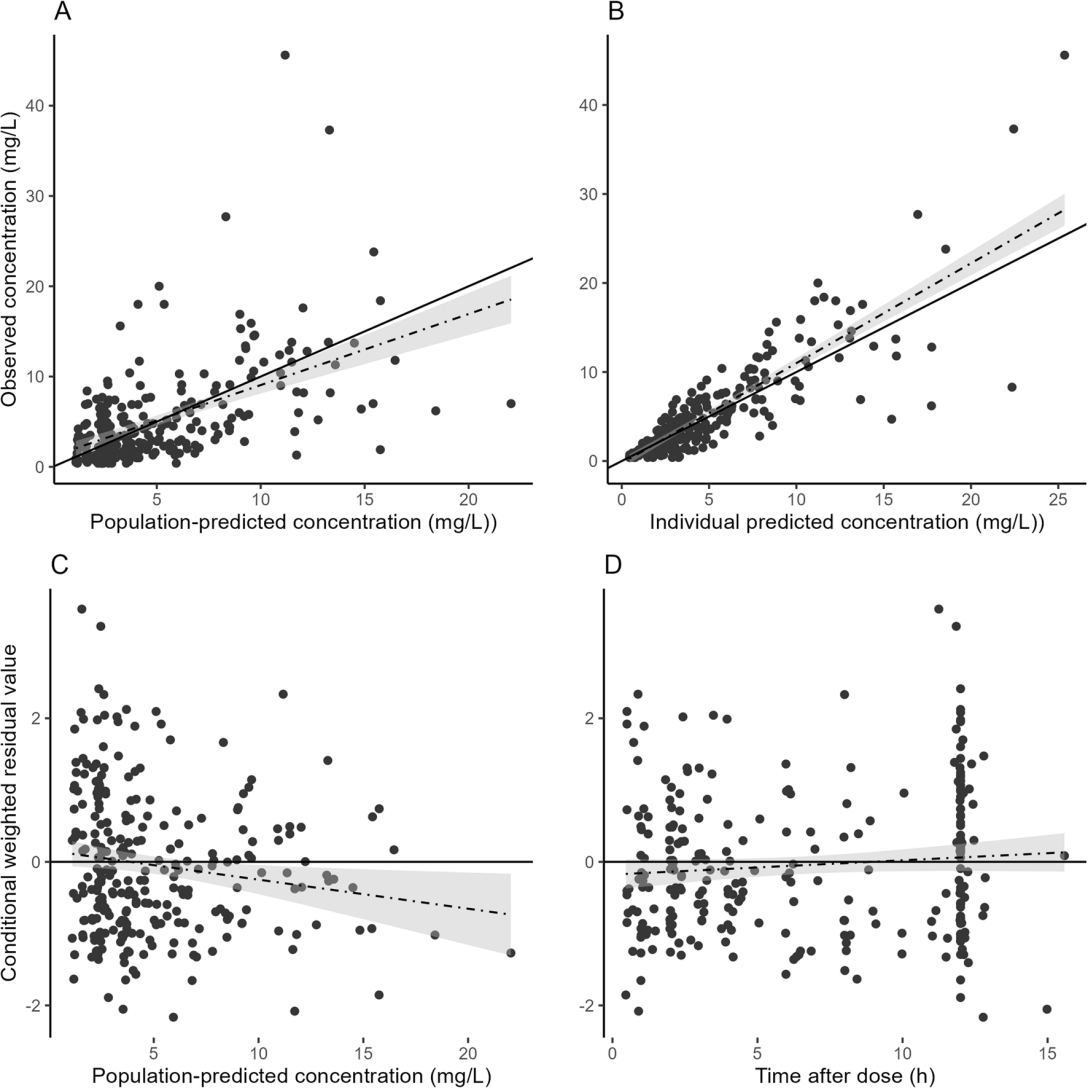
Goodness-of-fit plots for the final PK model on the model development dataset. **A** Model-predicted versus observed concentrations obtained for the final model based on estimated population parameters. **B** Model-predicted versus observed concentrations obtained for the final model based on estimated individual parameters. The solid line represents the line of identity; the dotted line, the linear regression line, and the grey fields represent the 95% CI. **C** Conditional weighted residuals (CWRES) vs. population prediction. **D** CWRES vs. time after dose

**Fig. 4:**
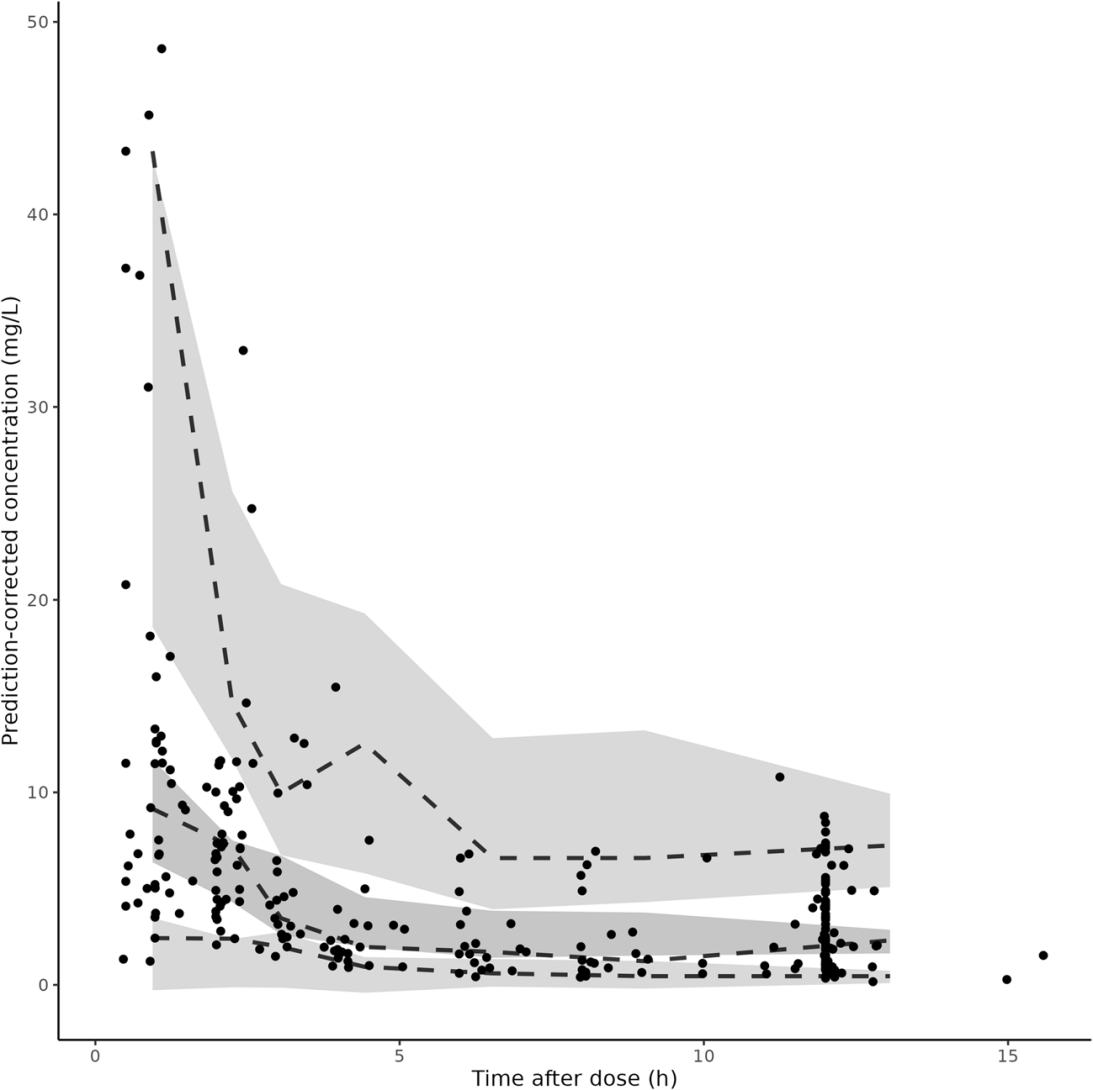
Visual predictive check on the model development dataset. The black dots denote the (prediction-corrected) observed concentrations. The lines represent the median and the 5th and 95th percentiles of the observed plasma concentrations. The 95% confidence intervals for the median, 5th and 95th percentiles of the model predictions are shown as grey areas

Figures 3 and 4 show the prediction-corrected visual predictive check and goodness-of-fit plots on the model development dataset. The population-predicted and individual-predicted MPA concentrations were similar to the observed data as evidenced by the consistent distributions around the line of identity (Fig. 3). The conditional weighted residuals exhibited a homogeneous distribution over the whole population’s predicted concentration range and sampling period (Fig. 3). In the prediction-corrected visual predictive check (Fig. 4), the percentiles of the observed concentrations were within the model-derived prediction intervals for these percentiles.

### External evaluation of the developed model

**Fig. 5:**
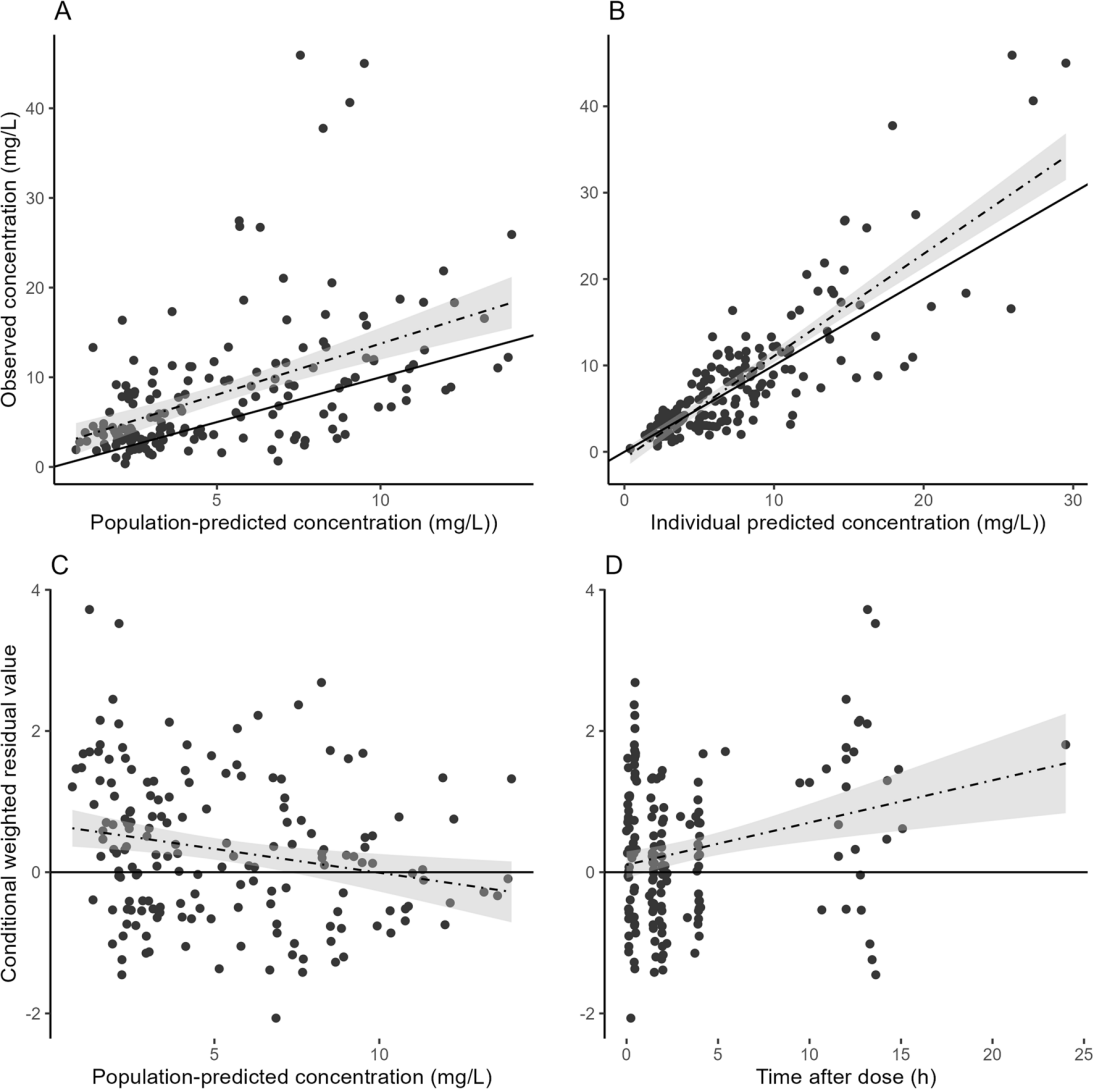
Goodness-of-fit plots of the final PK model on the external dataset. **A** Model-predicted versus observed concentrations obtained for the final model based on estimated population parameters. **B** Model-predicted versus observed concentrations obtained for the final model based on estimated individual parameters. The solid line represents the line of identity; the dotted line, the linear regression line, and the grey fields represent the 95% CI. **C** Conditional weighted residuals (CWRES) vs. population prediction. **D** CWRES vs. time after dose

The goodness-of-fit plots on the external dataset are presented in Fig. 5. The prediction-corrected visual check is presented in the supplementary material. As seen in the goodness-of-fit plots, the performance of the PK model in the external patient population was adequate, although with slight underprediction on the population level (Fig. 5), that can also be observed in the prediction-corrected visual check. However, the model performed well on an individual level, as no notable underprediction could be observed in the individual predicted versus the observed MPA concentrations (Fig. 5). This implies that our model may also be valid in other populations to aid dose optimization using individually measured MPA concentrations.

### Development of an improved starting dose

**Fig. 6:**
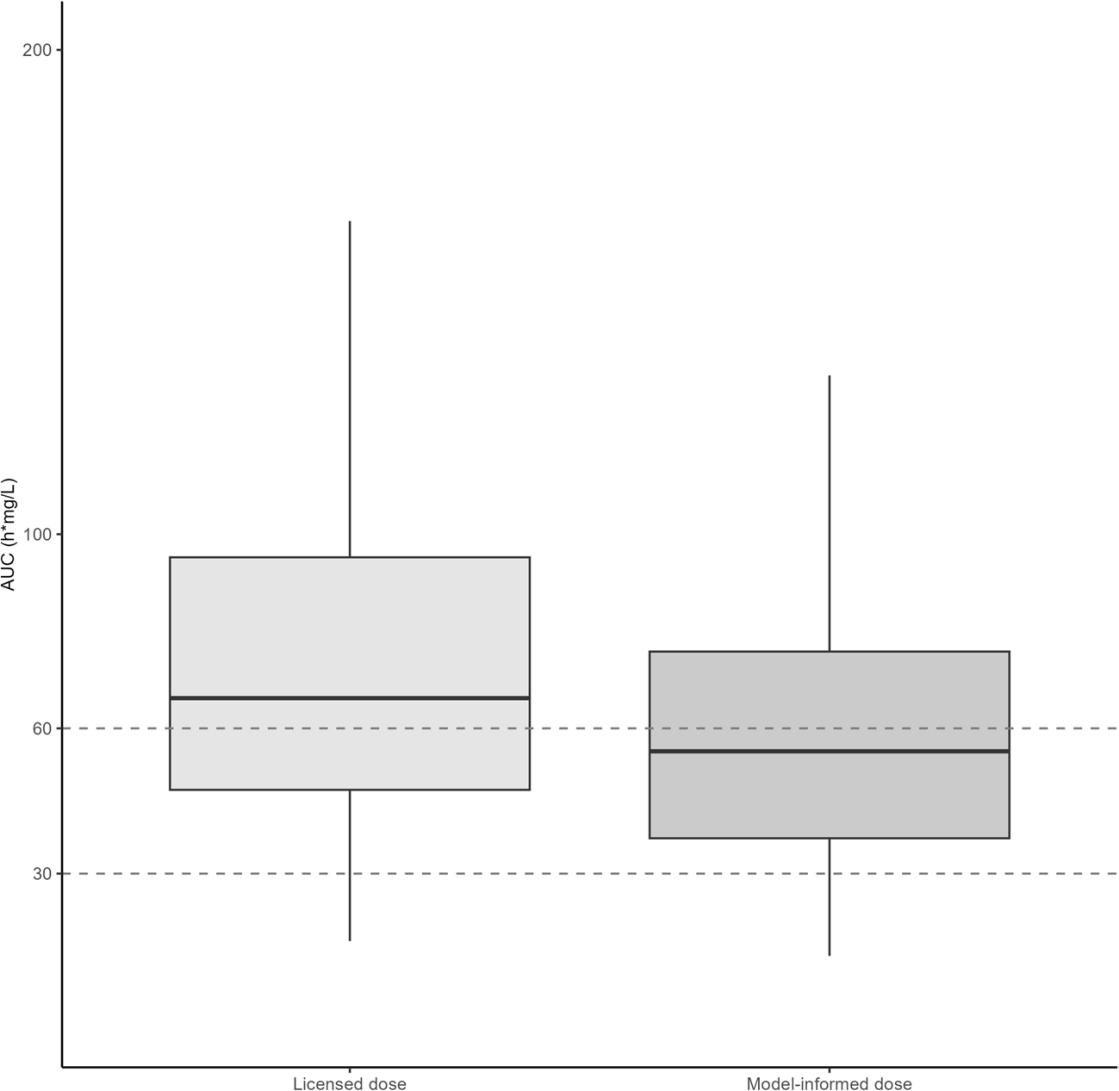
Box‐and‐whisker‐plot of the predicted AUC_0–12 h_ when using the dosing chart compared to the licensed dose. The dotted lines represent the target range of the AUC_0–12 h_. The bold horizontal bars in the middle show the median values, whereas the outer boundaries of the boxes represent the ranges of the 25th and 75th percentiles

The optimized starting dose scheme based on weight is presented in Table 3. Figure 6 shows that PK variability can be reduced and pharmacokinetic target attainment can be improved using the optimized starting dose. When using the licensed dose, 35% of the of the simulated population reached the desired target range without TDM-based dose adjustment. When using the optimized MMF starting dose, 42% of the population is predicted to reach the target AUC without TDM-based dose adjustment. Furthermore, when applying the optimized dosing scheme (Table 3), the median predicted AUC falls within the desired range of 30–60 mg h L^−1^. In contrast, the median predicted AUC when using the licensed dose lies above the target range.

**Table 3:** Optimized MMF starting dose for pediatric patients based on weight and albumin. Doses are described as twice the daily dose in milligrams

Weight (kg)	Twice daily dose (mg)
< 20	250
20–39	500
40–59	750
60–90	1000

## Discussion

In this study, we have successfully developed a PK model for MPA. MPA concentrations were well described by first-order elimination with Erlang-type absorption including one transition compartment, parameterized as K_tr_ (1.48 h^−1^), CL/F (16.0 L h^−1^), V_c_/F (24.9L), V_p_/F 1590 L), and Q/F (36.2 L h^−1^).

Five models have been previously published that describe the population PK of MPA in pediatric kidney transplant patients [2, 28–31]. Premaud et al. and Payen et al. described the PK of patients using ciclosporin as comedication, which is known to inhibit the enterohepatic circulation of MPA [2, 30]. In the study of Zhao et al., half of the patients also used ciclosporin as comedication [2, 28, 30]. The population parameters from these studies cannot be directly compared with our data because the unit of dose in our dataset was described in terms of MMF equivalents (milligrams of MMF) rather than MPA, as in the other studies. When parameters are corrected to MPA by multiplying the estimates with 0.739 (molecular weight MPA divided by molecular weight MMF), the estimated population values of MPA CL/F, V_c_/F, and Q/F in our model were generally well-aligned with previous studies [1]. However, the V_p_/F estimation of 1590 L was higher than previously reported values of 16.8 to 411.0 L in pediatric patients [1]. Before using this model for MIPD in clinical practice, evaluation of the current model and models in the literature is necessary to select the best-performing model.

We evaluated the model’s suitability to use for MIPD using a dataset containing limited sampling curves of pediatric kidney transplant patients from Erasmus MC. The model showed an underprediction on population level, yet adequate fit on the individual level. Differences in patient characteristics between the model development dataset and the external dataset such as post-transplant time, albumin level, and possibly comedication might explain that the model is not fully able to predict the PK of MPA in the external dataset on population level. As TDM dose adjustments are based on the individual fit of the model, it may be argued that our model could be used for MIPD in a clinical setting. This requires prospective evaluation.

Different methods were used to determine the MPA concentrations in the model-development data set and the external data set. The MPA concentration measurements for the model-development dataset have all been performed with a Cobas EMIT assay. The concentrations of the external evaluation dataset have been measured with either HPLC or Dade EMIT assay. A concentration-dependent overestimation of MPA concentrations by EMIT (both Cobas and Dade) has been reported in the literature compared to HPLC assays [3, 32–36]. This overestimation is due to cross-reactivity with the active acyl-glucuronide metabolite. This overestimation is especially apparent early after transplantation, as impaired renal function leads to elevated acyl-glucuronide metabolite levels [34, 37]. We here show that despite the possible discrepancy, our model is able to adequately capture MPA concentrations on an individual level, implying that our model may also be used for pharmacokinetically-guided dosing in other pediatric populations or other institutions using a different bioanalytical assay. Before the use of the model in institutions with a different analytical assay, we propose that this should first be prospectively evaluated.

Albumin was identified as a covariate in our model. The correlation between MPA clearance and albumin concentration might be explained through MPA plasma protein binding. MPA is highly bound to plasma proteins, and it was previously shown that low serum albumin decreases MPA protein binding [38]. Consequently, the unbound fraction increases, resulting in a decrease in total (bound and unbound) concentrations, explained by increased apparent total clearance in the model. Models of MPA for pediatric patients in literature did not include albumin as potential covariate. Either this was not investigated [2, 28, 30] or no significant relationship between albumin and apparent MPA clearance was found [29, 31].

We have proposed a dosing algorithm of MMF for pediatric patients based on body weight. The use of this algorithm for the initial MMF dose calculation immediately after transplantation can improve target attainment early after the start of treatment. These results may be the basis for moving to a more individualized, model-based dosing regimen. The achievement of therapeutic MPA levels early after transplantation could decrease the risk of rejection and reduce toxicities. When using the proposed improved dosing regimen, the AUC distribution was narrower and the MPA exposure of a higher proportion of patients was within the target range. As shown in Fig. 6, the optimized dosing strategy still leads to some supratherapeutic exposure. Nonetheless, we prefer to have a slightly elevated AUC to mitigate the risk of kidney rejection and the possibility of adjustment by TDM rather than starting with a subtherapeutic exposure.

Some limitations should be noted. One limitation is that our PK model did not include enterohepatic recirculation in the final model, even though a significant amount of MPA can be reabsorbed through this mechanism [39]. Other models describing MPA PK in the pediatric population have also not included the enterohepatic circulation. Enterohepatic circulation is difficult to capture in a model since it appears at unpredictable time intervals. The modelling of the enterohepatic circulation may improve the fitting of data exhibiting secondary peaks to allow a more accurate and precise estimation of MPA exposure. Therefore, the performance of our model to reliably estimate the AUC should be evaluated. This was beyond the scope of our current study. Another limitation is the use of retrospective PK data from routine care, potentially leading to inaccuracies in the recording of sampling times. Furthermore, a very high inter-individual variability for the central volume of distribution with a relatively large uncertainty margin was estimated. It may be debated whether this is true variability in volume of distribution or an artifact of variable absorption and enterohepatic circulation during the absorption phase. Since the AUC, the primary PK parameter used for dose individualization, is not affected by the individual estimate for volume, we postulate that this does not relevantly impact our findings on the optimal dose. Again, a prospective fit-for-purpose evaluation of our model to guide MPA dosing in pediatric renal transplant patients.

The population model reported in this study described the MPA PK in pediatric kidney transplant patients receiving a combination regimen of MMF and tacrolimus or everolimus and might not be extrapolated to an immunosuppressive regimen such as MMF in combination with cyclosporin, due to the known PK interaction [40, 41]. However, the combination of MMF and tacrolimus is the standard of care at the moment.

In conclusion, a population PK model was developed to describe the PK of MPA in pediatric kidney transplant recipients. Based on this model, we have developed a starting dose scheme for MMF that is expected to improve target attainment early in treatment. This dosing algorithm is a simple and feasible approach that could be implemented in clinical practice. Furthermore, the developed model will facilitate future studies on guiding MPA treatment using a model-based TDM strategy.

## Supplementary Information

Below is the link to the electronic supplementary material.

Supplementary file1 (DOCX 15 KB)

Supplementary file2 (DOCX 211 KB)

## Data Availability

The data that support the findings of this study are available from the corresponding author upon reasonable request.
