# Supplementary material for: Model-informed dose optimization of mycophenolic acid in pediatric kidney transplant patients
Source: Eur J Clin Pharmacol. 2024 Aug 17;80(11):1761–71. doi: 10.1007/s00228-024-03743-0 (PMC11458656; doi:10.1007/s00228-024-03743-0)
Supplement: Supplementary file 1 — Supplementary file1 (DOCX 15 KB) [file 228_2024_3743_MOESM1_ESM.docx]

**Supplementary Information**

Model-informed dose optimization of mycophenolic acid in pediatric kidney transplant patients

European Journal of Clinical Pharmacology

Astrid Heida^1^, Nynke G L Jager^1^, Rob Aarnoutse^1^, Brenda C M de Winter^2^, Huib de Jong^3^, Ron J Keizer^4^, Elisabeth A M Cornelissen^5^, Rob ter Heine^1^

1 Department of Pharmacy, Radboud Institute for Medical Innovation, Radboud university medical center, Nijmegen, The Netherlands

2 Department of Hospital Pharmacy, Erasmus University Medical Center, Rotterdam, The Netherlands.

3 The Erasmus MC Transplant Institute, Erasmus University Medical Center, Rotterdam, The Netherlands

4 Insight Rx, San Francisco, California, USA

5 Department of Pediatric Nephrology, Radboud university medical center, Amalia Children's Hospital, Nijmegen, The Netherlands

Corresponding author:

Astrid Heida

Department of Pharmacy, Radboud Institute for Medical Innovation, Radboud university medical center, Nijmegen, The Netherlands

E-mail address: Astrid.Heida@radboudumc.nl

**Model code**

$SUBROUTINES ADVAN5

$MODEL

COMP=(DOSE)

COMP=(TRAN)

COMP=(CENTRAL)

COMP=(PERIPHERAL)

$PK

; Time after dose needed for creating VPCs

IF (AMT.GT.0) THEN

TDOS=TIME

TAD=0.0

ENDIF

IF (AMT.EQ.0) TAD=TIME-TDOS

IF (OCC.EQ.1) IOV=ETA(6)

IF (OCC.EQ.2) IOV=ETA(7)

IF (OCC.EQ.3) IOV=ETA(8)

IF (OCC.EQ.4) IOV=ETA(9)

IF (OCC.EQ.5) IOV=ETA(10)

IF (OCC.EQ.6) IOV=ETA(11)

IF (OCC.EQ.7) IOV=ETA(12)

IF (OCC.EQ.8) IOV=ETA(13)

IF (OCC.EQ.9) IOV=ETA(14)

IF (OCC.EQ.10) IOV=ETA(15)

ALLOCL=(WT/70)**0.75

ALLOV=(WT/70)

ALLOK=(WT/70)**(-0.25)

COVALB=(ALB/34)**THETA(6)

CL=THETA(1)*ALLOCL*COVALB*EXP(ETA(1))

V2=THETA(2)*ALLOV*EXP(ETA(2))

V3=THETA(3)*ALLOV*EXP(ETA(3))

Q=THETA(4)*ALLOCL*EXP(ETA(4))

KTR=THETA(5)*ALLOK*EXP(ETA(5))

F1=1*EXP(IOV)

S2=V2

K12=KTR

K23=KTR

K34=Q/V3

K43=Q/V4

K30=CL/V3

$ERROR

IPRED=F

Y=IPRED+IPRED*ERR(1)

AUC=AMT/CL

$THETA

16.0 ;Cl

24.9 ;V3

1590 ;V4

36.2;Q

1.48 ; KA

-2.49; ALB~CL

$OMEGA

0.139; 1 IIV CL

2.42 ; 2 IIV V3

0 FIX ; 3 IIV V4

0.337 ; 4 IIV Q

0 FIX; 6 IVV KA

$OMEGA BLOCK(1) 0.19 ; OCC 1

$OMEGA BLOCK(1) SAME ; OCC 2

$OMEGA BLOCK(1) SAME ; OCC 3

$OMEGA BLOCK(1) SAME ; OCC 4

$OMEGA BLOCK(1) SAME ; OCC 5

$OMEGA BLOCK(1) SAME ; OCC 6

$OMEGA BLOCK(1) SAME ; OCC 7

$OMEGA BLOCK(1) SAME ; OCC 8

$OMEGA BLOCK(1) SAME ; OCC 9

$OMEGA BLOCK(1) SAME ; OCC 10

$SIGMA

0.223;PROP ERR

$ESTIMATION METHOD=1 NSIG=2 INTERACTION MAXEVAL=2000 NOHABORT

$COV PRINT=E MATRIX=S UNCONDITIONAL
