# Supplementary material for: Model-informed dose optimization of mycophenolic acid in pediatric kidney transplant patients
Source: Eur J Clin Pharmacol. 2024 Aug 17;80(11):1761–71. doi: 10.1007/s00228-024-03743-0 (PMC11458656; doi:10.1007/s00228-024-03743-0)
Supplement: Supplementary file 2 — Supplementary file2 (DOCX 211 KB) [file 228_2024_3743_MOESM2_ESM.docx]

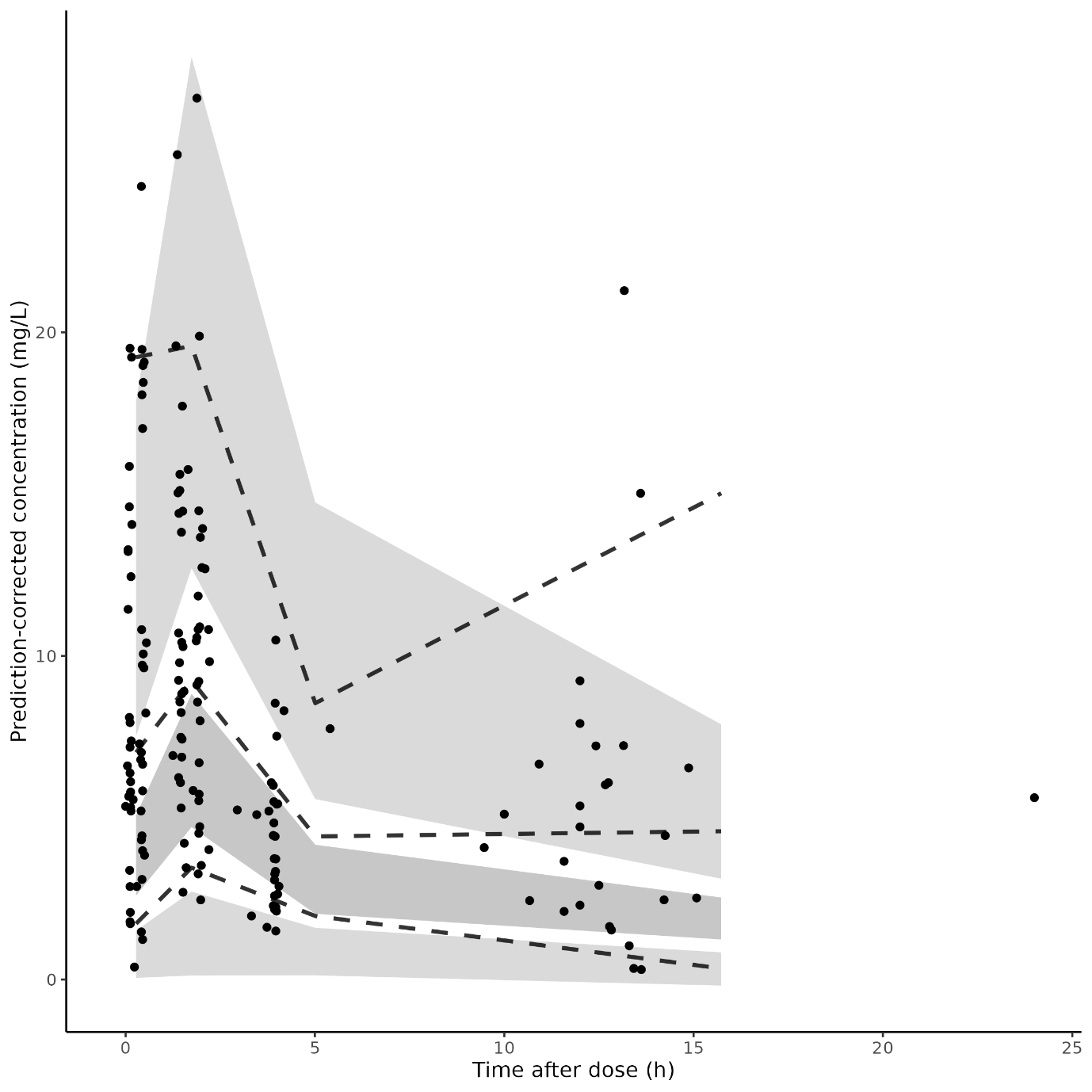


Figure S2. Visual predictive check on the external dataset. The black dots denote the observations. The lines represent the median and the 5th and 95th percentiles of the observed plasma concentrations. The median, 5th and 95th percentiles and the 95% confidence intervals for the corresponding model predicted percentiles are shown as grey fields.
